# Placebo Response and Its Predictors in Attention Deficit Hyperactivity Disorder: A Meta-Analysis and Comparison of Meta-Regression and MetaForest

**DOI:** 10.1093/ijnp/pyab054

**Published:** 2021-08-06

**Authors:** Xavier Castells, Marc Saez, Maghie Barcheni, Ruth Cunill, Domènec Serrano, Beatriz López, Caspar J van Lissa

**Affiliations:** 1 TransLab Research Group, Universitat de Girona, Girona, Spain; 2 Department of Medical Sciences, Universitat de Girona, Girona, Spain; 3 Economy Department; Research Group on Statistics, Econometrics and Health (GRECS), Universitat de Girona, Girona, Spain; 4 Centre for Biomedical Research Network in Epidemiology and Public Health (CIBERESP), Madrid, Spain; 5 Pharmacology, Toxicology and Therapeutics Department, Universitat Autònoma de Barcelona, Barcelona, Spain; 6 Parc Sanitari Sant Joan de Déu-Numància, Parc Sanitari Sant Joan de Déu, Barcelona, Spain; 7 Institut d’Assistència Sanitària, Girona, Spain; 8 Control and Intelligent Systems Engineering Research Group, Electrical; Electronic and Automatic Engineering Department, Universitat de Girona, Girona, Spain (Dr López); 9 Department of Methodology and Statistics, Universiteit Utrecht, Utrecht, The Netherlands (Dr van Lissa)

**Keywords:** Attention Deficit Hyperactivity Disorder, machine learning, MetaForest, meta-analysis, meta-regression, placebo response

## Abstract

**Background:**

High placebo response in attention deficit hyperactivity disorder (ADHD) can reduce medication–placebo differences, jeopardizing the development of new medicines. This research aims to (1) determine placebo response in ADHD, (2) compare the accuracy of meta-regression and MetaForest in predicting placebo response, and (3) determine the covariates associated with placebo response.

**Methods:**

A systematic review with meta-analysis of randomized, placebo-controlled clinical trial investigating pharmacological interventions for ADHD was performed. Placebo response was defined as the change from baseline in ADHD symptom severity assessed according to the 18-item, clinician-rated, DSM-based rating scale. The effect of study design–, intervention–, and patient–related covariates in predicting placebo response was studied by means of meta-regression and MetaForest.

**Results:**

Ninety-four studies including 6614 patients randomized to placebo were analyzed. Overall, placebo response was −8.9 points, representing a 23.1% reduction in the severity of ADHD symptoms. Cross-validated accuracy metrics for meta-regression were R^2^ = 0.0012 and root mean squared error = 3.3219 for meta-regression and 0.0382 and 3.2599 for MetaForest. Placebo response among ADHD patients increased by 63% between 2001 and 2020 and was larger in the United States than in other regions of the world.

**Conclusions:**

Strong placebo response was found in ADHD patients. Both meta-regression and MetaForest showed poor performance in predicting placebo response. ADHD symptom improvement with placebo has markedly increased over the last 2 decades and is greater in the United States than the rest of the world.

**Significance Statement**

In this article, we report the results of a systematic review and meta-analysis investigating placebo response in Attention Deficit Hyperactivity Disorder (ADHD). We included 94 randomized, placebo-controlled clinical trials involving 6614 patients. Overall placebo response was −8.9 points, representing a 23.1% reduction in the severity of ADHD symptoms. The effect of study design–, intervention–, and patient–related factors in predicting placebo response was studied by means of 2 different statistical methods: meta-regression and MetaForest. We found that meta-regression and MetaForest had low accuracy in predicting placebo response. Finally, placebo response among ADHD patients increased by 63% between 2001 and 2020 and was larger in the United States than in other regions of the world. To the best of our knowledge, this is the first study to apply MetaForest in pharmacology research to date and to compare meta-regression and MetaForest performance in predicting placebo response.

## Introduction

Randomized, placebo-controlled clinical trial (RPCCT) is the gold standard method for determining the efficacy of therapeutic interventions. New medicines must demonstrate a suitable benefit-risk relationship in RPCCTs to gain marketing authorization from the Food and Drug Administration (in the United States) and the European Medicines Agency (in the European Union). However, RPCCTs have a high failure rate in psychiatry (Nutt and Goodwin, 2011) and neurology (Alphs et al., 2012; Benedetti et al., 2016), diminishing the likelihood of new medicines becoming available. Because high placebo response can reduce medication–placebo differences, the large placebo response in RPCCTs of psychiatric medications (Walsh et al., 2002; Vieta and Cruz, 2008; Agid et al., 2013; Rutherford et al., 2014) might be an important factor in the high RPCCT failure rate. To overcome this reduction in medication–placebo differences, multi-center trials are usually needed. However, these trials are both complex and expensive.

The increasing complexity of clinical trials in psychiatry, alongside the number of failed trials in recent years, jeopardizes research and development of psychiatric drugs as they have become more expensive and time-consuming compared with medications for non-central nervous system indications (Nutt and Goodwin, 2011). Unsurprisingly, several pharmaceutical companies have reduced or discontinued research and development of medications for brain disorders (Kesselheim et al., 2015), prompting warnings of “psychopharmacology in crisis” (Stahl and Greenberg, 2019).

An understanding of placebo response in RPCCTs could aid in optimizing RPCCT design and reducing the likelihood of effective treatments being erroneously deemed as ineffective. It could further help decrease the number of patients exposed to placebo to a minimum, which has important ethical implications (Miller and Colloca, 2011).

Comprehensive analysis of placebo response and the factors influencing placebo response in RPCCTs has been conducted for schizophrenia, depression, and mania (Walsh et al., 2002; Khan et al., 2003; Papakostas and Fava, 2009; Yildiz et al., 2011; Undurraga and Baldessarini, 2012; Nierenberg et al., 2015; Welten et al., 2015; Leucht et al., 2018; Fraguas et al., 2019). In contrast, few studies have investigated placebo response in attention deficit hyperactivity disorder (ADHD) (Newcorn et al., 2009; Waschbusch et al., 2009; Waxmonsky et al., 2011; Buitelaar et al., 2012; Khan et al., 2017; Cohen et al., 2018; Ben-Sheetrit et al., 2020). This gap is relevant because ADHD is a prevalent neurodevelopmental disorder (Thomas et al., 2015) that has significant clinical and psychosocial consequences (Daley and Birchwood, 2010; Charach et al., 2011; Klein et al., 2012; Dalsgaard et al., 2015). Moreover, pharmacological treatment, alongside psychosocial support, is considered a cornerstone in the management of ADHD (Banaschewski et al., 2018; National Institute for Health and Clinical Excellence (NICE), 2018; The Canadian ADHD Resource Allieance (CADDRA), 2020; Faraone et al., 2021).

Many RPCCTs investigating the efficacy of medications for ADHD have been conducted (Cunill et al., 2016; Riera et al., 2017; Cortese et al., 2018). In this context, a systematic review with meta-analysis is considered one of the most reliable methods for making sense of data from different studies. However, no systematic review of placebo response in ADHD has yet been published. Only 1 meta-analysis, which was limited to 17 RPCCTs submitted to the Food and Drug Administration between 2000 and 2009, has studied placebo response in patients with ADHD (Khan et al., 2017). Newcorn and colleagues (Newcorn et al., 2009) reported a pooled analysis of 10 acute RPCCT of atomoxetine for ADHD, Waxmonsky (Waxmonsky et al., 2011) and Buitelaar (Buitelaar et al., 2012) reported 2 secondary analyses of the placebo response in 2 RPCCTs of lisdexamfetamine and osmotic-release oral system methylphenidate, respectively, and Ben-Sheerit (Ben-Sheetrit et al., 2020) studied the placebo response using data from 1 RPCCT investigating metadoxine. Therefore, a comprehensive systematic review of placebo response in ADHD is lacking.

A systematic review with meta-analysis of the RPCCT investigating the efficacy of ADHD medications would allow for combining the results of different studies, calculating an overall placebo response and determining between-study variability or heterogeneity. The standard approach to analyzing the sources of between-study variability is meta-regression. Meta-regression quantifies the influence of moderators of the intervention effect size. Nevertheless, meta-regression is prone to overfitting (the model explains heterogeneity of the observed data but does not generalize well to new data; see (Van Lissa, 2020) when either too many moderators are included in the model or when the selection of the moderators included in the final meta-regression model is made after preforming multiple meta-regression analyses, using forward or backward stepwise selection. Furthermore, moderator analysis is compromised when there is multicollinearity between moderators (Terrer et al., 2019).

MetaForest is a machine learning method that applies the random forest algorithm to meta-analysis. For investigating between-study variability, MetaForest has some advantages over meta-regression, namely, it is robust to overfitting, captures non-linear relationships, and performs variable selection (Van Lissa, 2017). MetaForest has previously been used in climate research (Terrer et al., 2019) and research on early-life stress in animal models (Bonapersona et al., 2019).

This study aims to (1) determine placebo response in RPCCTs investigating the efficacy of ADHD drugs, (2) compare the performance of meta-regression and MetaForest in predicting placebo response, and (3) determine the patient-, intervention-, and study design–related covariates associated with placebo response. To the best of our knowledge, this is the first study to apply MetaForest in pharmacology research to date and to compare meta-regression and MetaForest performance in predicting placebo response.

## METHODS

This project was made reproducible using the Workflow for Open Reproducible Code in Science (Van Lissa et al., 2020). The data and analysis code are available online at https://github.com/cjvanlissa/placebo_adhd.

### Design and Inclusion/Exclusion Criteria

We conducted a systematic review with meta-analysis of RPCCTs performed in an outpatient setting, investigating the efficacy of pharmacological interventions for ADHD irrespective of their approval status. To be included, RPCCTs had to assess the efficacy on ADHD symptoms using an 18-item, clinician-rated, DSM-based ADHD rating scale scoring from 0 to 54 points. The length of the intervention had to be at least 2 weeks. We excluded studies with a lead-in phase, those in which an additional psychological intervention for ADHD or a psychopharmacological intervention is administered, those investigating efficacy maintenance, and those published only as a conference abstracts.

### Data Source

Data were obtained from the Minerva database (https://minervadatabase.org/en) on June, 15, 2020 (Minerva Database, 2020). The Minerva database contains comprehensive information on randomized controlled trials that have investigated the efficacy and safety of pharmacological interventions for ADHD. These RPCCTs were identified using systematic search techniques on multiple information sources: Medline, Cochrane CENTRAL, Psycinfo, clinicaltrials.gov, clinicaltrialsregister.eu, and controlled-trials.com. Through a system of weekly alerts, the contents of the Minerva database are updated each time new studies are identified. At the time of the search, Minerva database stored data from 322 randomized, controlled studies. The information stored in the Minerva database includes administrative information, study methods, patient characteristics, study results from each clinical trial, and risk of bias of each study and outcome. Minerva database has been used in previous studies demonstrating its utility (Castells et al., 2020b, 2020a).

### Study Variables

The primary outcome of interest was the placebo response assessed using the 18-item, clinician-rated, DSM IV, IV-TR, or 5-based ADHD rating scale. Each item corresponds to 1 of the 18 DSM IV, IV-TR, or DSM-5 criteria. The severity for each item is rated on a 4-point scale (from 0 to 3), resulting in a total score range from 0 to 54 points, with higher scores denoting more severe symptoms. Because placebo response is defined as the change from baseline in ADHD symptom severity, it usually takes negative values, with lower scores indicating greater symptom improvement. By using similar investigator-rated scales that have the same scoring, we can express the results in the natural scale of the instrument.

For the second and third study objectives, the independent variables were study design–, patient–, and intervention–related characteristics. Specifically, the study design–related variables were: study design (parallel vs cross-over); treatment naïve as an inclusion criterion; comorbidity as an inclusion criterion, number of study sites, probability of receiving placebo (calculated as N randomized to placebo/N randomized to any study intervention), and type of analysis (intention to treat: yes/no). Patient-related variables were mean age, sex (% of men), illness severity at baseline, and ethnicity (% Caucasian). Intervention-related variables were type of drug (psychostimulant: yes/no), legal status of the drug (approved for ADHD vs not approved for ADHD), drug dosage (fixed vs variable), treatment duration, and concomitant psychotherapy. Other variables were publication year, region (USA/others), sponsorship, and risk of bias using the Cochrane risk of bias tool (yes/no) (Higgins et al., 2011). Covariates with more than 50% of missing data were removed.

### Procedures

Using random sampling, the study database was split into a training set (70%) and a test set. Meta-regression and MetaForest models were trained using the “training dataset,” and the “test dataset” was used to compare their accuracy in predicting placebo response. The most accurate method in the previous analysis was applied to the whole data set to determine overall placebo response and the covariates associated with placebo response.

### Statistical Analysis

First, placebo response was determined by pooling the mean change of ADHD symptom severity in the placebo group of each included study using a random effect model (DerSimonian and Laird, 1986). Statistical heterogeneity was assessed with the uncertainty factor I^2^, which measures the percentage of variance across studies due to heterogeneity rather than chance (Higgins and Thompson, 2002). Publication bias was studied with the funnel plot and the Egger test (Egger et al., 1997).

Before performing meta-regression and MetaForest, the presence of multicollinearity was examined using the generalized variance inflation factor (Fox and Monette, 1992). If collinearity was present, several meta-regression models were generated by removing each collinear covariate once and comparing them using the likelihood ratio method. Covariates found to be not relevant were withdrawn. Afterwards, missing data were imputed using Multiple Imputation by Chained Equations (Azur et al., 2011; Doove et al., 2014; Shah et al., 2014).

Meta-regression was performed as follows. First, we performed a univariate method of moments-based meta-regression of each potential study moderator. Those covariates with a *P* < .1 were included in the multivariable meta-regression model. The statistical significance was set at *P* < .05 in the multivariate model.

MetaForest was performed following the methods described elsewhere (Van Lissa, 2020). First, the number of trees required for convergence was determined. Then, variables were preselected by replicating the analysis 100 times and retaining variables with positive variable importance in >95% of replications applied. Finally, the MetaForest hyperparameters (number of variables to consider at each split, minimum number of studies in each node, and study weighting using uniform, fixed-effect, or random-effects weights) were tuned with leave-one-out cross-validation using the caret package (see public code for further details: https://github.com/cjvanlissa/placebo_adhd).

Finally, the predictive performance of meta-regression and MetaForest performance was compared. The models estimated on the “training dataset” were used to predict placebo response of the studies available in the “test dataset.” The resulting predicted responses were compared with the actual placebo response using the root mean squared error (RMSE) and the R^2^. The method showing the smallest RMSE and the largest R^2^ was considered the most accurate.

### Study Registration

The study protocol was registered on the International Prospective Register of Systematic Reviews: CRD42020196738 (https://www.crd.york.ac.uk/prospero/display_record.php?RecordID=196738).

## RESULTS

### Study Characteristics

Ninety-four studies were included, which randomized 6614 patients to placebo (see supplementary Figure 1 and Table 2 for flow diagram and study references, respectively). We did not exclude any covariate due to insufficient information. Imputation of missing data yielded similar values to the observed ones (see supplementary Figure 3 for density plots). No multicollinearity of covariates was found and thus no covariate was deemed irrelevant.

**Table 2.** Analysis of the Covariates Associated With Placebo Response Using Random Effects Multivariate Meta-Regression on the Whole Dataset

	Effect (SE)	*P* value
Intercept	15.45 (7.49)	.0392
Design (parallel)	−0.99 (1.35)	.4633
Number of centers	−0.06 (0.02)	.0007
Concomitant psychotherapy administered (yes)	−3.33 (2.06)	.1054
Publication date (y)	−0.22 (0.07)	.0010
Study location (USA)	−2.39 (0.90)	.0081
Sponsor (pharmaceutical industry)	5.08 (1.46)	.0005
	tau = 2.53 I^2^ = 78.13%; R^2^ = 46.02%	

Studies and patients’ characteristics are reported in Table 1. Most studies had a parallel design, were multicenter, and conducted the statistical analysis using an intention to treat approach. To be drug naïve and to have a comorbid psychiatric disorder were infrequent inclusion criteria. Overall, the mean age was 22 years, two-thirds of patients were male, and most were Caucasian and had moderate to severe ADHD. The probability of receiving placebo was approximately 1:3. The most frequently studied interventions were non-stimulants and drugs approved for the treatment of ADHD. Study medication was administered at fixed dosages in almost one-half of the studies. Length of treatment was relatively short, averaging 8 weeks. Concomitant psychotherapy was infrequently administered. Almost one-half of the studies were published in the 21st century, and the majority were conducted in the USA and had a commercial sponsor. Approximately one-quarter had a high risk of bias, and high patient dropout rate was the most frequent reason for deeming studies to have a “high” risk of bias.

**Table 1.** Study Characteristics^*a*^

Study design–related covariates	
Design (% parallel)	92.6
Naïve as inclusion criterion (% yes)	8.5
Comorbidity as an inclusion criterion (%)	14.9
Number of centres (mean, range)	23 (1–71)
Probability of receiving placebo (mean, range)	38.4 (10.8–58.6)
ITT analysis (%)	80.9
Patient-related covariates	
Age (mean, range)	22.2 (5.1–41.4)
Sex (% men, range)	64.6 (27.7–100)
Race (% Caucasian, range)	71.2 (0–100)
Mean baseline ADHD severity (mean, range)	38.6 (30.4–46.9)
Intervention-related covariates	
Type of drug (% psychostimulant)	35.1
Approval status (% drug approved for treating ADHD)	75.5
Dosificaction (% fixed dosification)	45.7
Treatment length (wk) (mean, range)	8 (2–28)
Concomitant psychotherapy administered (% with psychotherapy)	3.2
Other covariates	
Publication date (%)	
2001–2010	46.8
2011–2020	53.2
Study location (% USA)	86.2
Sponsor (% pharmaceutical industry)	92.6
Risk of bias (% high risk)	24.5

Abbreviations: ADHD, Attention Deficit Hyperactivity Disorder, ITT, intention to treat, USA, United States of America, wk, weeks.

^*a*^Patient-level binary variables are expressed as percent and range, and study-level variables as percent.

### Objective 1: Placebo Response

**Figure 1.**
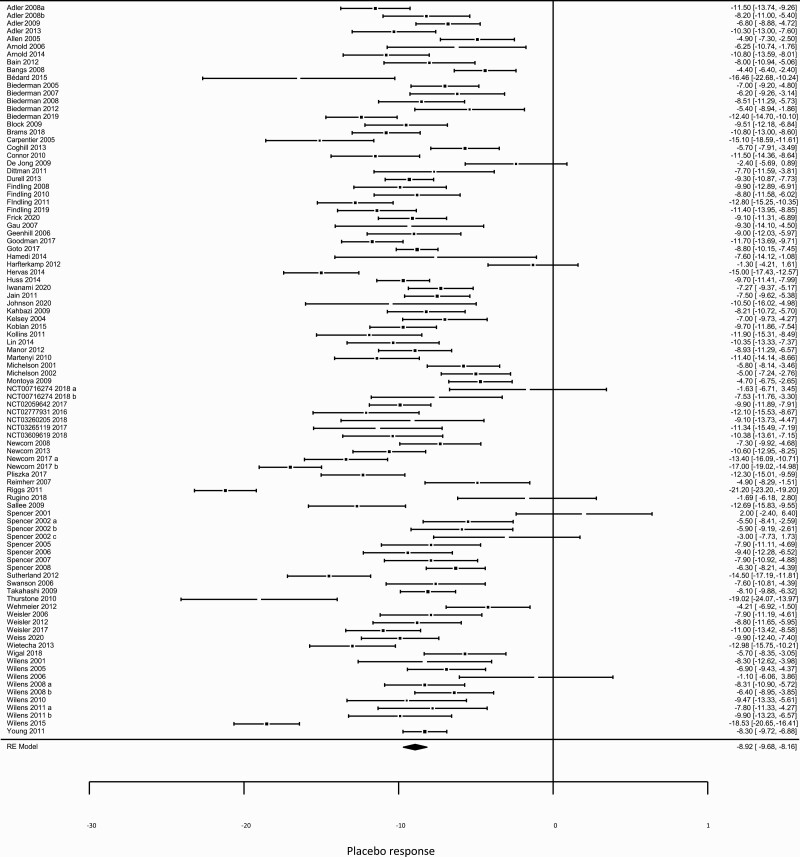
Forest plot of placebo response in a randomized, placebo-controlled clinical trial (RPCCT) of pharmacological interventions for attention deficit hyperactivity disorder (ADHD).

The pooled placebo response, using the whole dataset, was 8.9, representing an overall estimated 23.1% reduction in the severity of ADHD symptoms (Figure 1). Statistical heterogeneity in placebo response was large, as shown by an I^2^ = 86.67%, indicating that 86.67% of variance in placebo response was due to between-study heterogeneity rather than to sampling error. There was no evidence of publication bias based on an acceptably symmetrical funnel plot (supplementary Figure 4) and non-statistically significant Egger’s test.

### Objective 2: Comparison of Meta-Regression and MetaForest Performance

Dataset splitting yielded 2 sets of studies with similar characteristics (descriptive statistics of the training and testing samples are displayed in supplementary Table 5). The univariate meta-regression using the training dataset (supplementary Table 6) identified design, number of centers, concomitant psychotherapy, publication date, and sponsor as potentially relevant explanatory covariates, which were included in the multivariate meta-regression model. This analysis found that placebo response was influenced by publication date and sponsor (supplementary Table 7). This model explained 47.29% of variance in the training data.

Using the training dataset, MetaForest analysis found that design, race, publication date, and study location were the most relevant covariates. The analysis was replicated and retained only 10 covariates (see supplementary Tables 8 and 9).

The predictive performance of meta-regression and MetaForest was estimated using the test dataset. The comparison between the predicted and the actual results showed that both models had poor generalizability, as indicated by small R^2^ and RMSE: 0.0012 and 3.3219 for meta-regression and 0.0382 and 3.2599 for MetaForest.

### Objective 3: Covariates Associated With Placebo Response

Because no clear differences in performance between meta-regression and MetaForest were found, both methods were used to determine the influence of study covariates on placebo response using the whole dataset. The univariate meta-regressions analysis identified study design, number of centers, concomitant psychotherapy administered, publication date, study location, and sponsor as potentially relevant explanatory covariates (supplementary Table 10) and were included in the multivariate meta-regression model (Table 2). This analysis found that placebo response was influenced by the number of centers, publication date, study location, and sponsor. This model explained 46% of variance.

**Figure 2.**
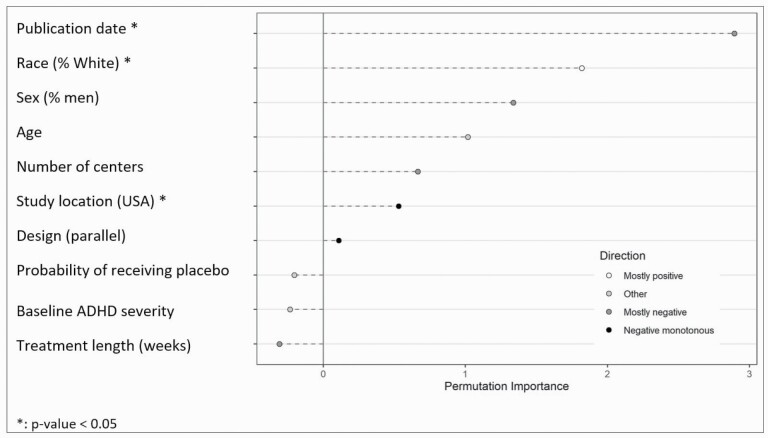
Summary of the results of the MetaForest analysis using the whole dataset.

Figure 2 shows a summary of the results of the MetaForest analysis (with additional results in supplementary Table 11). This analysis found publication date, race, and study location to be the most relevant covariates.

**Figure 3.**
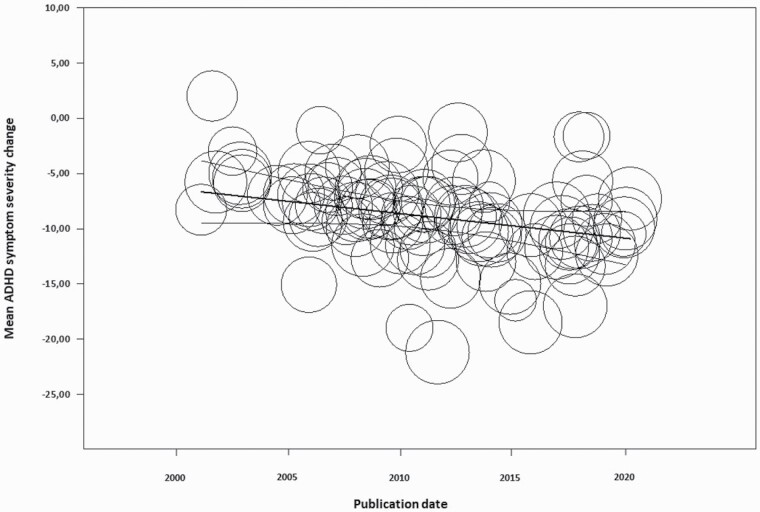
Scatter plot of the effect of publication date on placebo response adjusted for study design, number of centers, concomitant psychotherapy administered, study location, and sponsor.

Publication date was found to be a relevant covariate in both the meta-regression and MetaForest analyses on both the training and the whole datasets. Figure 3 shows that placebo response increased from −6.7 in 2001 to −10.9 in 2020.

## Discussion

We analyzed 94 studies that randomized 6614 patients with ADHD to placebo and found that placebo had a significant effect. The reduction of almost 9 points in ADHD symptom severity compared with baseline corresponds to a 23% symptom reduction, which is not far from the 25% to 30% threshold considered as a clinically relevant improvement of ADHD symptoms in many RPCCTs (Michelson et al., 2002; Spencer et al., 2002; Medori et al., 2008; Weisler et al., 2012; Philipsen et al., 2015; Johnson et al., 2020; Saito et al., 2020). Given that blinding is likely to be compromised in RPCCT due to the behavioral effects of the studied medications, it is possible that placebo response is higher than we have found. Our findings are consistent with previous research on placebo response in patients with ADHD using non-systematic review methodology (Newcorn et al., 2009; Waschbusch et al., 2009; Waxmonsky et al., 2011; Buitelaar et al., 2012; Khan et al., 2017; Cohen et al., 2018; Ben-Sheetrit et al., 2020).

It is difficult to specify the clinical implications of this finding because participating in an RPCCT involves a higher number of clinical visits, more time spent with the physician and research staff, and a higher number of tests performed than in the clinical practice. Therefore, it is unlikely that administering placebo in the clinical setting will result in symptom improvement as observed in the context of RPCCTs.

Placebo response has been documented in other psychiatric disorders such as depression (Undurraga and Baldessarini, 2012), acute schizophrenia (Leucht et al., 2018), stable schizophrenia (Fraguas et al., 2019), bipolar mania (Yildiz et al., 2011; Welten et al., 2015), bipolar depression (Bridge et al., 2009; Nierenberg et al., 2015), and obsessive compulsive disorder (Ackerman and Greenland, 2002; Kotzalidis et al., 2018). Unlike mania, depression, and acute schizophrenia, where severity of symptoms change over a short period of time, ADHD is a relatively stable condition and the reduction of ADHD symptoms during the clinical trial is less likely to be due to the natural course of the disorder. Instead, expectations may play a major role in placebo response in patients with ADHD. Patients and particularly clinicians may expect symptom improvement in the context of an RPCCT, because several pharmacological interventions have consistently demonstrated their ability to reduce ADHD symptom severity and are recommended as first-line treatment (Bolea-Alamañac et al., 2014).

Large statistical heterogeneity was observed. The causes of such heterogeneity were investigated by means of meta-regression and MetaForest. Both methods showed poor performance because models estimated on the training dataset did not generalize to the testing dataset. This means that, although our models describe a significant proportion of the variance in the training data (e.g., meta-regression described almost 50% of statistical heterogeneity), the patterns do not generalize to the test data. This lack of generalizability implies that we cannot predict the placebo response of future studies from our results. Based on these results, it is not possible to tailor the design of RPCCTs to achieve low placebo response and increase the odds of detecting medication–placebo differences. The contrast between variance explained in the training data vs the testing data is an important reminder that meta-regression models may (severely) overfit. For this reason, we recommend that model performance on a testing set should be determined as routine practice when investigating the sources of between-study variability. This quality control is seldom performed, and carrying it out would provide valuable information on the external validity of the study findings.

Publication date was found to influence placebo response in all analyses. Specifically, placebo response in ADHD increased by 63% between 2001 and 2020. An increase of placebo response has been documented before by Khan et al. (2017) in a meta-analysis of 17 clinical trials of ADHD medications approved by the US Food and Drug Administration between 2000 and 2009 as well as in other fields of psychiatry such as schizophrenia (Agid et al., 2013; Leucht et al., 2018), depression (Walsh et al., 2002; Papakostas and Fava, 2009; Undurraga and Baldessarini, 2012), obsessive compulsive disorder (Ackerman and Greenland, 2002; Kotzalidis et al., 2018), and bipolar disorder (Sysko and Walsh, 2007). Changes over time in baseline severity (Bridge et al., 2009; Nierenberg et al., 2015; Cohen et al., 2018), number of study centers (Bridge et al., 2009; Yildiz et al., 2011; Undurraga and Baldessarini, 2012; Agid et al., 2013; Leucht et al., 2018; Fraguas et al., 2019), sex (Yildiz et al., 2011; Welten et al., 2015), study quality (Agid et al., 2013), and type of drug (Agid et al., 2013) have frequently been alluded to as an explanation for this time-related increase of placebo response in psychiatry. These covariates do not seem to confound the moderating effect of publication date in our study because none of them were found to be associated with placebo response in any analysis. We speculate that, as public awareness of the efficacy of pharmacological interventions for ADHD has increased with time, patients’ and clinicians’ expectations of treatment efficacy may have increased from 2001 to 2020, leading to increased placebo response.

We also found study location to be associated with placebo response in the meta-regression and MetaForest analyses using the whole dataset. Placebo response was 2.4 points higher in the United States than in the rest of the world. Because ADHD is more frequently diagnosed and treated in the United States than in the rest of the world and research is also more prevalent in this country, expectations regarding the efficacy of pharmacological treatment may also be higher, resulting in higher placebo response. Nevertheless, it must be stressed that some studies on disorders other than ADHD have found placebo response to be lower in the United States (Mallinckrodt et al., 2010; Welten et al., 2015; Leucht et al., 2018, 2019), and other studies have found no effect of location on placebo response (Bridge et al., 2009; Agid et al., 2013). Altogether, these results suggest that the true effect of location might be small or near zero and, for this reason, some studies find a positive, others a negative, and others a null effect.

The probability of receiving placebo was not found to be a moderator of placebo response. This is notable because it is one of the covariates most frequently associated with placebo response (Papakostas and Fava, 2009; Mallinckrodt et al., 2010; Agid et al., 2013; Nierenberg et al., 2015; Fraguas et al., 2019). Our findings should, however, be interpreted with caution because we did not investigate whether the lack of association between the probability of receiving placebo and placebo response was confounded by the effect of other covariates in the meta-regression analysis.

Our previous research on the sources of between-study variability in the efficacy and safety of pharmacological treatment for ADHD found treatment length, type of drug, comorbidity as inclusion criteria, and sponsorship as moderators (Cunill et al., 2016; Riera et al., 2017). None of these covariates has been found to moderate placebo response in the current study. Others have reported similar discrepancies between moderators of efficacy and of placebo response (Leucht et al., 2019). This indicates that results of placebo response cannot be straightforwardly extrapolated to treatment efficacy.

### Limitations

The main limitation of this research is that the number of studies included may be insufficient to obtain adequate power for investigating sources of between-study variability and comparing model performance. Power calculations for meta-analyses are complex and require assumptions about moderator effect sizes. This is at odds with the present exploratory approach to moderator analysis. The primary implication of this limitation is that the results may have limited generalizability to future studies, but they do serve as a description of the present literature. A second limitation is that, although we have included most covariates investigated in other studies, some important covariates may have been omitted, for example, illness duration (Agid et al., 2013), which is usually not reported in ADHD trials. Third, as with any study dealing with aggregated data, the possibility of ecological bias must always be taken into consideration when interpreting the findings of this study (Greenland and Morgenstern, 1989). Fourth, some categories (e.g., region or comorbidities) show skewed distribution. To cope with class imbalance, we grouped studies into broader categories such as “not US” or “comorbidity as an inclusion criterion,” which facilitates the statistical analysis at the expense of reducing the amount of information analyzed. Fifth, we classified modafinil and bupropion as non-stimulants. This decision is arguable because they are chemically related to methylphenidate and amphetamines and have mild psychostimulant effects (Schmitt and Reith, 2012; Chevassus et al., 2013). Sixth, to better understand placebo response, it would be necessary to compare the change in ADHD symptoms between patients receiving placebo and those receiving no intervention. Nevertheless, no study included in our meta-analysis has such design. Finally, we have only investigated placebo response on ADHD symptom severity as assessed by clinicians. Therefore, the possibility that placebo response differs when symptoms are assessed by teachers, patients, or parents remains to be investigated.

## Conclusions

In spite of these limitations, we can conclude that:

- Notable improvement in ADHD symptoms was found with the administration of placebo;
- Despite describing almost 50% of variance in the training data, meta-regression and MetaForest performed poorly in predicting placebo responses in the testing dataset;
- Model performance should be routinely assessed to provide information regarding the validity of the results;
- ADHD symptom improvement with placebo has increased over the past 2 decades and is greater in the United States than in the rest of the world.

## Supplementary Material

pyab054_suppl_Supplementary_Materials

## Interest Statement

Xavier Castells is the manager of the Minerva Database. Domènec Serrano has received speaker fees from Janssen-Cilag.
