# Supplementary material for: Placebo Response and Its Predictors in Attention Deficit Hyperactivity Disorder: A Meta-Analysis and Comparison of Meta-Regression and MetaForest
Source: Int J Neuropsychopharmacol. 2021 Aug 6;25(1):26–35. doi: 10.1093/ijnp/pyab054 (PMC8756096; doi:10.1093/ijnp/pyab054)
Supplement: pyab054_suppl_Supplementary_Materials [file pyab054_suppl_supplementary_materials.docx]

**Online supplementary material**

**Index**

[**Supplementary Figure S1: Flow diagram of the selection of studies** 2](#_Toc63111299)

[**Supplementary Table S2: References of the studies** **included** 3](#_Toc63111304)

[**Supplementary Figure S3: Density plots** 11](#_Toc63111305)

[**Supplementary Figure S4: Funnel plot** 12](#_Toc63111306)

[**Supplementary Table S5: Comparison of the distribution of the study outcome and covariates between the train and the test dataset** 13](#_Toc63111307)

[**Supplementary Table S6: Univariate meta-regression results using the training dataset** 15](#_Toc63111308)

[**Supplementary Table S7: Multivariate meta-regression results using the training dataset** 16](#_Toc63111309)

[**Supplementary Table S8: MetaForest analysis results using the training dataset** 17](#_Toc63111310)

[**Supplementary Table S9: Summary of the results of the MetaForest analysis using the training dataset** 18](#_Toc63111311)

[**Supplementary Table S10: Univariate meta-regression using a random effects model using the whole dataset** 19](#_Toc63111312)

[**Supplementary Table S11: Summary of the results of the MetaForest analysis using the whole dataset** 20](#_Toc63111313)

# **Supplementary Figure S1: Flow diagram of the selection of studies**

Date: June 15^th^ 2020

**Supplementary Table S2: References of the studies included**

Full-text records assessed for eligibility
(n = 1,417)

## Identification

Studies meeting inclusion/exclusion criteria

(n = 94)

(n = 102)

Studies included in Minerva

(n = 322)

Additional records identified through other sources
(n = 74)

Records identified through database searching
(n = 6262)

## Screening

## Included

Records excluded
(n = 4919)

## Eligibility

Studies excluded
(n = 228)

Records screened
(n = 6,336)

Full-text records excluded, with reasons
(not a randomized controlled clinical trial investigating the efficacy/safety of pharmacological interventions for ADHD)

(n = 668)

| **Study** | **References** |
| --- | --- |
| Adler 2008a | Adler LA, Spencer TJ, Levine LR, Ramsey JL, Tamura R, Kelsey D, Ball SG, Allen AJ, Biederman J (2008) Functional outcomes in the treatment of adults with ADHD. J Atten Disord 11:720–727 |
| Adler 2008b | Adler LA, Goodman DW, Kollins SH, Weisler RH, Krishnan S, Zhang Y, Biederman J (2008) Double-blind, placebo-controlled study of the efficacy and safety of lisdexamfetamine dimesylate in adults with attention-deficit/hyperactivity disorder. J Clin Psychiatry 69:1364–1373 |
| Adler 2009 | Adler LA, Zimmerman B, Starr HL, Silber S, Palumbo J, Orman C, Spencer T (2009) Efficacy and safety of OROS methylphenidate in adults with attention-deficit/hyperactivity disorder: a randomized, placebo-controlled, double-blind, parallel group, dose-escalation study. J Clin Psychopharmacol 29:239–247 |
| Adler 2013 | Adler LA, Dirks B, Deas PF, Raychaudhuri A, Dauphin MR, Lasser RA, Weisler RH (2013) Lisdexamfetamine dimesylate in adults with attention-deficit/hyperactivity disorder who report clinically significant impairment in executive function: Results from a randomized, double-blind, placebo-controlled study. J Clin Psychiatry 74:694–702 |
| Allen 2005 | Allen AJ, Kurlan RM, Gilbert DL, Coffey BJ, Linder SL, Lewis DW, Winner PK, Dunn DW, Dure LS, Sallee FR, Milton DR, Mintz MI, Ricardi RK, Erenberg G, Layton LL, Feldman PD, Kelsey DK, Spencer TJ (2005) Atomoxetine treatment in children and adolescents with ADHD and comorbid tic disorders. :1941–1949 |
| Arnold 2006 | Arnold LE, Aman MG, Cook AM, Witwer AN, Hall KL, Thompson S, Ramadan Y (2006) Atomoxetine for Hyperactivity in Autism Spectrum Disorders: Placebo-Controlled Crossover Pilot Trial. J Am Acad Child Adolesc Psychiatry 45:1196–1205 |
| Arnold 2014 | Arnold VK, Feifel D, Earl CQ, Yang R, Adler L a (2014) A 9-week, randomized, double-blind, placebo-controlled, parallel-group, dose-finding study to evaluate the efficacy and safety of modafinil as treatment for adults with ADHD. J Atten Disord 18:133–144 |
| Bain 2012 | Bain EE, Apostol G, Sangal RB, Robieson WZ, McNeill DL, Abi-Saab WM, Saltarelli MD (2012) A randomized pilot study of the efficacy and safety of ABT-089, a novel α4β2 neuronal nicotinic receptor agonist, in adults with attention-deficit/hyperactivity disorder. J Clin Psychiatry 73:783–789 |
| Bangs 2008 | Bangs ME, Hazell P, Danckaerts M, Hoare P, Coghill DR, Wehmeier PM, Williams DW, Moore RJ, Levine L (2008) Atomoxetine for the treatment of attention-deficit/hyperactivity disorder and oppositional defiant disorder. Pediatrics 121:e314-20 |
| Bédard 2015 | Bédard A-C V, Schulz KP, Krone B, Pedraza J, Duhoux S, Halperin JM, Newcorn JH (2015) Neural mechanisms underlying the therapeutic actions of guanfacine treatment in youth with ADHD: a pilot fMRI study. Psychiatry Res 231:353–356 |
| Biederman 2005 | Biederman J (2005) Efficacy and Safety of Modafinil Film-Coated Tablets in Children and Adolescents With Attention-Deficit/Hyperactivity Disorder: Results of a Randomized, Double-Blind, Placebo-Controlled, Flexible-Dose Study. Pediatrics 116:e777–e784 |
| Biederman 2007 | Biederman J, Krishnan S, Zhang Y, McGough JJ, Findling RL (2007) Efficacy and tolerability of lisdexamfetamine dimesylate (NRP-104) in children with attention-deficit/hyperactivity disorder: a phase III, multicenter, randomized, double-blind, forced-dose, parallel-group study. Clin Ther 29:450–463 |
| Biederman 2008 | Biederman J, Melmed RD, Patel A, McBurnett K, Konow J, Lyne A, Scherer N (2008) A randomized, double-blind, placebo-controlled study of guanfacine extended release in children and adolescents with attention-deficit/hyperactivity disorder. Pediatrics 121:e73-84 |
| Biederman 2012 | Biederman J, Fried R, Hammerness P, Surman C, Mehler B, Petty CR, Faraone S V, Miller C, Bourgeois M, Meller B, Godfrey KM, Reimer B (2012) The effects of lisdexamfetamine dimesylate on the driving performance of young adults with ADHD: a randomized, double-blind, placebo-controlled study using a validated driving simulator paradigm. J Psychiatr Res 46:484–491 |
| Biederman 2019 | Biederman J, Lindsten A, Sluth LB, Petersen ML, Ettrup A, Eriksen H-LF, Fava M (2019) Vortioxetine for attention deficit hyperactivity disorder in adults: A randomized, double-blind, placebo-controlled, proof-of-concept study. J Psychopharmacol 33:511–521 |
| Block 2009 | Block SL, Kelsey D, Coury D, Lewis D, Quintana H, Sutton V, Schuh K, Allen AJ, Sumner C (2009) Once-daily atomoxetine for treating pediatric attention-deficit/hyperactivity disorder: comparison of morning and evening dosing. Clin Pediatr (Phila) 48:723–733 |
| Brams 2018 | Brams M, Childress AC, Greenbaum M, Yu M, Yan B, Jaffee M, Robertson B (2018) SHP465 Mixed Amphetamine Salts in the Treatment of Attention-Deficit/Hyperactivity Disorder in Children and Adolescents: Results of a Randomized, Double-Blind Placebo-Controlled Study. J Child Adolesc Psychopharmacol 28:19–28 |
| Carpentier 2005 | Carpentier PJ, De Jong CAJ, Dijkstra BAG, Verbrugge CAG, Krabbe PFM (2005) A controlled trial of methylphenidate in adults with attention deficit/hyperactivity disorder and substance use disorders. Addiction 100:1868–1874 |
| Coghill 2013 | Coghill D, Banaschewski T, Lecendreux M, Soutullo C, Johnson M, Zuddas A, Anderson C, Civil R, Higgins N, Lyne A, Squires L (2013) European, randomized, phase 3 study of lisdexamfetamine dimesylate in children and adolescents with attention-deficit/hyperactivity disorder. Eur Neuropsychopharmacol J Eur Coll Neuropsychopharmacol 23:1208–1218 |
| Connor 2010 | Connor DF, Findling RL, Kollins SH, Sallee F, López FA, Lyne A, Tremblay G (2010) Effects of guanfacine extended release on oppositional symptoms in children aged 6-12 years with attention-deficit hyperactivity disorder and oppositional symptoms: a randomized, double-blind, placebo-controlled trial. CNS Drugs 24:755–768 |
| De Jong 2009 | de Jong CGW, Van De Voorde S, Roeyers H, Raymaekers R, Allen AJ, Knijff S, Verhelst H, Temmink AH, Smit LME, Rodriques-Pereira R, Vandenberghe D, van Welsen I, ter Schuren L, Al-Hakim M, Amin A, Vlasveld L, Oosterlaan J, Sergeant JA (2009) Differential effects of atomoxetine on executive functioning and lexical decision in attention-deficit/hyperactivity disorder and reading disorder. J Child Adolesc Psychopharmacol 19:699–707 |
| Dittman 2011 | Dittmann RW, Schacht A, Helsberg K, Schneider-Fresenius C, Lehmann M, Lehmkuhl G, Wehmeier PM (2011) Atomoxetine versus placebo in children and adolescents with attention-deficit/hyperactivity disorder and comorbid oppositional defiant disorder: a double-blind, randomized, multicenter trial in Germany. J Child Adolesc Psychopharmacol 21:97–110 |
| Durell 2013 | Durell TM, Adler L a, Williams DW, Deldar A, McGough JJ, Glaser PE, Rubin RL, Pigott T a, Sarkis EH, Fox BK (2013) Atomoxetine treatment of attention-deficit/hyperactivity disorder in young adults with assessment of functional outcomes: a randomized, double-blind, placebo-controlled clinical trial. J Clin Psychopharmacol 33:45–54 |
| Findling 2008 | Findling RL, Bukstein OG, Melmed RD, López FA, Sallee FR, Arnold LE, Pratt RD (2008) A randomized, double-blind, placebo-controlled, parallel-group study of methylphenidate transdermal system in pediatric patients with attention-deficit/hyperactivity disorder. J Clin Psychiatry 69:149–159 |
| Findling 2010 | Findling RL, Turnbow J, Burnside J, Melmed R, Civil R, Li Y (2010) A randomized, double-blind, multicenter, parallel-group, placebo-controlled, dose-optimization study of the methylphenidate transdermal system for the treatment of ADHD in adolescents. CNS Spectr 15:419–430 |
| FIndling 2011 | Findling RL, Childress AC, Cutler AJ, Gasior M, Hamdani M, Ferreira-Cornwell MC, Squires L (2011) Efficacy and safety of lisdexamfetamine dimesylate in adolescents with attention-deficit/hyperactivity disorder. J Am Acad Child Adolesc Psychiatry 50:395–405 |
| Findling 2019 | Findling RL, Adler LA, Spencer TJ, Goldman R, Hopkins SC, Koblan KS, Kent J, Hsu J, Loebel A (2019) Dasotraline in Children with Attention-Deficit/Hyperactivity Disorder: A Six-Week, Placebo-Controlled, Fixed-Dose Trial. J Child Adolesc Psychopharmacol 29:80–89 |
| Frick 2020 | Frick G, Yan B, Adler LA (2020) Triple-Bead Mixed Amphetamine Salts (SHP465) in Adults With ADHD: Results of a Phase 3, Double-Blind, Randomized, Forced-Dose Trial. J Atten Disord 24:402–413 |
| Gau 2007 | Gau SSF, Huang Y-S, Soong W-T, Chou M-C, Chou W-J, Shang C-Y, Tseng W-L, Allen AJ, Lee P (2007) A randomized, double-blind, placebo-controlled clinical trial on once-daily atomoxetine in Taiwanese children and adolescents with attention-deficit/hyperactivity disorder. J Child Adolesc Psychopharmacol 17:447–460 |
| Geenhill 2006 | Greenhill LL, Biederman J, Boellner SW, Rugino TA, Sangal RB, Earl CQ, Jiang JG, Swanson JM (2006) A Randomized, Double-Blind, Placebo-Controlled Study of Modafinil Film-Coated Tablets in Children and Adolescents With Attention-Deficit/Hyperactivity Disorder. J Am Acad Child Adolesc Psychiatry 45:503–511 |
| Goodman 2017 | Goodman DW, Starr HL, Ma Y-W, Rostain AL, Ascher S, Armstrong RB (2017) Randomized, 6-Week, Placebo-Controlled Study of Treatment for Adult Attention-Deficit/Hyperactivity Disorder: Individualized Dosing of Osmotic-Release Oral System (OROS) Methylphenidate With a Goal of Symptom Remission. J Clin Psychiatry 78:105–114 |
| Goto 2017 | Goto T, Hirata Y, Takita Y, Trzepacz PT, Allen AJ, Song D-H, Gau SS-F, Ichikawa H, Takahashi M (2017) Efficacy and Safety of Atomoxetine Hydrochloride in Asian Adults With ADHD. J Atten Disord 21:100–109 |
| Hamedi 2014 | Hamedi M, Mohammadi M, Ghaleiha A, Keshavarzi Z, Jafarinia M (2014) Bupropion in Adults with Attention-Deficit / Hyperactivity Disorder : a Randomized , Double-blind Study. Acta Med Iran 52:675–681 |
| Harfterkamp 2012 | Harfterkamp M, van de Loo-Neus G, Minderaa RB, van der Gaag R-J, Escobar R, Schacht A, Pamulapati S, Buitelaar JK, Hoekstra PJ (2012) A randomized double-blind study of atomoxetine versus placebo for attention-deficit/hyperactivity disorder symptoms in children with autism spectrum disorder. J Am Acad Child Adolesc Psychiatry 51:733–741 |
| Hervas 2014 | Hervas A, Huss M, Johnson M, McNicholas F, van Stralen J, Sreckovic S, Lyne A, Bloomfield R, Sikirica V, Robertson B (2014) Efficacy and safety of extended-release guanfacine hydrochloride in children and adolescents with attention-deficit/hyperactivity disorder: A randomized, controlled, Phase III trial. Eur Neuropsychopharmacol 24:1861–1872 |
| Huss 2014 | Huss M, Ginsberg Y, Tvedten T, Arngrim T, Philipsen A, Carter K, Chen C-W, Kumar V (2014) Methylphenidate hydrochloride modified-release in adults with attention deficit hyperactivity disorder: a randomized double-blind placebo-controlled trial. Adv Ther 31:44–65 |
| Iwanami 2020 | Iwanami A, Saito K, Fujiwara M, Okutsu D, Ichikawa H (2020) Efficacy and Safety of Guanfacine Extended-Release in the Treatment of Attention-Deficit/Hyperactivity Disorder in Adults: Results of a Randomized, Double-Blind, Placebo-Controlled Study. J Clin Psychiatry 81:19m12979 |
| Jain 2011 | Jain R, Segal S, Kollins SH, Khayrallah M (2011) Clonidine extended-release tablets for pediatric patients with attention-deficit/hyperactivity disorder. J Am Acad Child Adolesc Psychiatry 50:171–179 |
| Johnson 2020 | Johnson JK, Liranso T, Saylor K, Tulloch G, Adewole T, Schwabe S, Nasser A, Findling RL, Newcorn JH (2020) A Phase II Double-Blind, Placebo-Controlled, Efficacy and Safety Study of SPN-812 (Extended-Release Viloxazine) in Children With ADHD. J Atten Disord 24:348–358 |
| Kahbazi 2009 | Kahbazi M, Ghoreishi A, Rahiminejad F, Mohammadi M-R, Kamalipour A, Akhondzadeh S (2009) A randomized, double-blind and placebo-controlled trial of modafinil in children and adolescents with attention deficit and hyperactivity disorder. Psychiatry Res 168:234–237 |
| Kelsey 2004 | Kelsey DK (2004) Once-Daily Atomoxetine Treatment for Children With Attention-Deficit/Hyperactivity Disorder, Including an Assessment of Evening and Morning Behavior: A Double-Blind, Placebo-Controlled Trial. Pediatrics 114:e1–e8 |
| Koblan 2015 | Koblan KS, Hopkins SC, Sarma K, Jin F, Goldman R, Kollins SH, Loebel A (2015) Dasotraline for the Treatment of Attention-Deficit/Hyperactivity Disorder: A Randomized, Double-Blind, Placebo-Controlled, Proof-of-Concept Trial in Adults. Neuropsychopharmacol 40:2745–2752 |
| Kollins 2011 | Kollins SH, López F a, Vince BD, Turnbow JM, Farrand K, Lyne A, Wigal SB, Roth T (2011) Psychomotor functioning and alertness with guanfacine extended release in subjects with attention-deficit/hyperactivity disorder. J Child Adolesc Psychopharmacol 21:111–120 |
| Lin 2014 | Lin DY, Kratochvil CJ, Xu W, Jin L, D’Souza DN, Kielbasa W, Allen AJ (2014) A randomized trial of edivoxetine in pediatric patients with attention-deficit/hyperactivity disorder. J Child Adolesc Psychopharmacol 24:190–200 |
| Manor 2012 | Manor I, Ben-Hayun R, Aharon-Peretz J, Salomy D, Weizman A, Daniely Y, Megiddo D, Newcorn JH, Biederman J, Adler LA (2012) A randomized, double-blind, placebo-controlled, multicenter study evaluating the efficacy, safety, and tolerability of extended-release metadoxine in adults with attention-deficit/hyperactivity disorder. J Clin Psychiatry 73:1517–1523 |
| Martenyi 2010 | Martenyi F, Zavadenko NN, Jarkova NB, Yarosh A a, Soldatenkova VO, Bardenstein LM, Kozlova I a, Neznanov NG, Maslova OI, Petrukhin AS, Sukchotina NK, Zykov VP (2010) Atomoxetine in children and adolescents with attention-deficit/hyperactivity disorder: a 6-week, randomized, placebo-controlled, double-blind trial in Russia. Eur Child Adolesc Psychiatry 19:57–66 |
| Michelson 2001 | Michelson D, Faries D, Wernicke J, Kelsey D, Kendrick K, Sallee FR, Spencer T (2001) Atomoxetine in the treatment of children and adolescents with attention-deficit/hyperactivity disorder: a randomized, placebo-controlled, dose-response study. Pediatrics 108:E83 |
| Michelson 2002 | Michelson D, Allen AJ, Busner J, Casat C, Dunn D, Kratochvil C, Newcorn J, Sallee FR, Sangal RB, Saylor K, West S, Kelsey D, Wernicke J, Trapp NJ, Harder D (2002) Once-daily atomoxetine treatment for children and adolescents with attention deficit hyperactivity disorder: A randomized, placebo-controlled study. Am J Psychiatry 159:1896–1901 |
| Montoya 2009 | Montoya A, Hervas A, Cardo E, Artigas J, Mardomingo MJ, Alda J a, Gastaminza X, García-Polavieja MJ, Gilaberte I, Escobar R (2009) Evaluation of atomoxetine for first-line treatment of newly diagnosed, treatment-naïve children and adolescents with attention deficit/hyperactivity disorder. Curr Med Res Opin 25:2745–2754 |
| NCT00716274 2018a | <https://clinicaltrials.gov/ct2/show/NCT00716274> |
| NCT00716274 2018b | <https://clinicaltrials.gov/ct2/show/NCT00716274> |
| NCT02059642 2017 | https://clinicaltrials.gov/ct2/show/NCT02059642 |
| NCT02777931 2016 | https://clinicaltrials.gov/ct2/show/NCT02777931 |
| NCT03260205 2018 | https://clinicaltrials.gov/ct2/show/NCT03260205 |
| NCT03265119 2017 | https://clinicaltrials.gov/ct2/show/NCT03265119 |
| NCT03609619 2018 | https://clinicaltrials.gov/ct2/show/NCT03609619 |
| Newcorn 2008 | Newcorn JH et al. (2008) Atomoxetine and osmotically released methylphenidate for the treatment of attention deficit hyperactivity disorder: Acute comparison and differential response. Am J Psychiatry 165:721–730 |
| Newcorn 2013 | Newcorn JH, Stein MA, Childress AC, Youcha S, White C, Enright G, Rubin J (2013) Randomized, double-blind trial of guanfacine extended release in children with attention-deficit/hyperactivity disorder: Morning or evening administration. J Am Acad Child Adolesc Psychiatry 52:921–930 |
| Newcorn 2017a and 2017b | Newcorn JH, Nagy P, Childress AC, Frick G, Yan B, Pliszka S (2017) Randomized, Double-Blind, Placebo-Controlled Acute Comparator Trials of Lisdexamfetamine and Extended-Release Methylphenidate in Adolescents With Attention-Deficit/Hyperactivity Disorder. CNS Drugs 31:999–1014 |
| Pliszka 2017 | Pliszka SR, Wilens TE, Bostrom S, Arnold VK, Marraffino A, Cutler AJ, López FA, DeSousa NJ, Sallee FR, Incledon B, Newcorn JH (2017) Efficacy and Safety of HLD200, Delayed-Release and Extended-Release Methylphenidate, in Children with Attention-Deficit/Hyperactivity Disorder. J Child Adolesc Psychopharmacol 27:474–482 |
| Reimherr 2007 | Reimherr FW, Williams ED, Strong RE, Mestas R, Soni P, Marchant BK (2007) A double-blind, placebo-controlled, crossover study of osmotic release oral system methylphenidate in adults with ADHD with assessment of oppositional and emotional dimensions of the disorder. J Clin Psychiatry 68:93–101 |
| Riggs 2011 | Riggs PD et al. (2011) Randomized controlled trial of osmotic-release methylphenidate with cognitive-behavioral therapy in adolescents with attention-deficit/hyperactivity disorder and substance use disorders. J Am Acad Child Adolesc Psychiatry 50:903–914 |
| Rugino 2018 | Rugino TA (2018) Effect on Primary Sleep Disorders When Children With ADHD Are Administered Guanfacine Extended Release. J Atten Disord 22:14–24 |
| Sallee 2009 | Sallee FR, Mcgough J, Wigal T, Donahue J, Lyne A, Biederman J (2009) Guanfacine Extended Release in Children and Adolescents With Attention-Deficit/Hyperactivity Disorder: A Placebo-Controlled Trial. J Am Acad Child Adolesc Psychiatry 48:155–165 |
| Spencer 2001 | Spencer T, Biederman J, Wilens T, Faraone S, Prince J, Gerard K, Doyle R, Parekh A, Kagan J, Bearman SK (2001a) Efficacy of a mixed amphetamine salts compound in adults with attention-deficit/hyperactivity disorder. Arch Gen Psychiatry 58:775–782 |
| Spencer 2002a and 2002b | Spencer T, Heiligenstein JH, Biederman J, Faries DE, Kratochvil CJ, Conners CK, Potter WZ (2002b) Results from 2 proof-of-concept, placebo-controlled studies of atomoxetine in children with attention-deficit/hyperactivity disorder. J Clin Psychiatry 63:1140–1147 |
| Spencer 2002c | Spencer T, Biederman J, Coffey B, Geller D, Crawford M, Bearman SK, Tarazi R, Faraone S V (2002a) A double-blind comparison of desipramine and placebo in children and adolescents with chronic tic disorder and comorbid attention-deficit/hyperactivity disorder. Arch Gen Psychiatry 59:649–656 |
| Spencer 2005 | Spencer T, Biederman J, Wilens T, Doyle R, Surman C, Prince J, Mick E, Aleardi M, Herzig K, Faraone S (2005) A large, double-blind, randomized clinical trial of methylphenidate in the treatment of adults with attention-deficit/hyperactivity disorder. Biol Psychiatry 57:456–463 |
| Spencer 2006 | Spencer TJ, Wilens TE, Biederman J, Weisler RH, Read SC, Pratt R (2006) Efficacy and safety of mixed amphetamine salts extended release (Adderall XR) in the management of attention-deficit/hyperactivity disorder in adolescent patients: a 4-week, randomized, double-blind, placebo-controlled, parallel-group study. Clin Ther 28:266–279 |
| Spencer 2007 | Spencer TJ, Adler LA, McGough JJ, Muniz R, Jiang H, Pestreich L (2007) Efficacy and Safety of Dexmethylphenidate Extended-Release Capsules in Adults with Attention-Deficit/Hyperactivity Disorder. Biol Psychiatry 61:1380–1387 |
| Spencer 2008 | Spencer TJ, Adler LA, Weisler RH, Youcha SH (2008) Triple-bead mixed amphetamine salts (SPD465), a novel, enhanced extended-release amphetamine formulation for the treatment of adults with ADHD: A randomized, double-blind, multicenter, placebo-controlled study. J Clin Psychiatry 69:1437–1448 |
| Sutherland 2012 | Sutherland SM, Adler L a, Chen C, Smith MD, Feltner DE (2012) An 8-week, randomized controlled trial of atomoxetine, atomoxetine plus buspirone, or placebo in adults with ADHD. J Clin Psychiatry 73:445–450 |
| Swanson 2006 | Swanson JM, Greenhill LL, Lopez FA, Sedillo A, Earl CQ, Jiang JG, Biederman J (2006) Modafinil film-coated tablets in children and adolescents with attention-deficit/hyperactivity disorder: results of a randomized, double-blind, placebo-controlled, fixed-dose study followed by abrupt discontinuation. J Clin Psychiatry 67:137–147 |
| Takahashi 2009 | Takahashi M, Takita Y, Yamazaki K, Hayashi T, Ichikawa H, Kambayashi Y, Koeda T, Oki J, Saito K, Takeshita K, Allen AJ (2009) A randomized, double-blind, placebo-controlled study of atomoxetine in Japanese children and adolescents with attention-deficit/hyperactivity disorder. J Child Adolesc Psychopharmacol 19:341–350 |
| Thurstone 2010 | Thurstone C, Riggs PD, Salomonsen-Sautel S, Mikulich-Gilbertson SK (2010) Randomized, controlled trial of atomoxetine for attention-deficit/hyperactivity disorder in adolescents with substance use disorder. J Am Acad Child Adolesc Psychiatry 49:573–582 |
| Wehmeier 2012 | Wehmeier PM, Schacht A, Ulberstad F, Lehmann M, Schneider-Fresenius C, Lehmkuhl G, Dittmann RW, Banaschewski T (2012) Does atomoxetine improve executive function, inhibitory control, and hyperactivity? Results from a placebo-controlled trial using quantitative measurement technology. J Clin Psychopharmacol 32:653–660 |
| Weisler 2006 | Weisler RH, Biederman J, Spencer TJ, Wilens TE, Faraone S V, Chrisman AK, Read SC, Tulloch SJ (2006) Mixed amphetamine salts extended-release in the treatment of adult ADHD: a randomized, controlled trial. CNS Spectr 11:625–639 |
| Weisler 2012 | Weisler RH, Pandina GJ, Daly EJ, Cooper K, Gassmann-Mayer C (2012) Randomized clinical study of a histamine H 3 receptor antagonist for the treatment of adults with attention-deficit hyperactivity disorder. CNS Drugs 26:421–434 |
| Weisler 2017 | Weisler RH, Greenbaum M, Arnold V, Yu M, Yan B, Jaffee M, Robertson B (2017) Efficacy and Safety of SHP465 Mixed Amphetamine Salts in the Treatment of Attention-Deficit/Hyperactivity Disorder in Adults: Results of a Randomized, Double-Blind, Placebo-Controlled, Forced-Dose Clinical Study. CNS Drugs 31:685–697 |
| Weiss 2020 | Weiss MD, Childress AC, Donnelly GAE (2020) Efficacy and Safety of PRC-063, Extended-Release Multilayer Methylphenidate in Adults with ADHD Including 6-Month Open-Label Extension. J Atten Disord:1087054719896853 |
| Wietecha 2013 | Wietecha L, Williams D, Shaywitz S, Shaywitz B, Hooper SR, Wigal SB, Dunn D, McBurnett K (2013) Atomoxetine improved attention in children and adolescents with attention-deficit/hyperactivity disorder and dyslexia in a 16 week, acute, randomized, double-blind trial. J Child Adolesc Psychopharmacol 23:605–613 |
| Wigal 2018 | Wigal TL, Newcorn JH, Handal N, Wigal SB, Mulligan I, Schmith V, Konofal E (2018) A Double-Blind, Placebo-Controlled, Phase II Study to Determine the Efficacy, Safety, Tolerability and Pharmacokinetics of a Controlled Release (CR) Formulation of Mazindol in Adults with DSM-5 Attention-Deficit/Hyperactivity Disorder (ADHD). CNS Drugs 32:289–301 |
| Wilens 2001 | Wilens TE, Spencer TJ, Biederman J, Girard K, Doyle R, Prince J, Polisner D, Solhkhah R, Comeau S, Monuteaux MC, Parekh A (2001b) A Controlled Clinical Trial of Bupropion for Attention Deficit Hyperactivity Disorder in Adults. :20–22 |
| Wilens 2005 | Wilens TE, Haight BR, Horrigan JP, Hudziak JJ, Rosenthal NE, Connor DF, Hampton KD, Richard NE, Modell JG (2005) Bupropion XL in adults with attention-deficit/hyperactivity disorder: a randomized, placebo-controlled study. Biol Psychiatry 57:793–801 |
| Wilens 2006 | Wilens TE, Verlinden MH, Adler LA, Wozniak PJ, West SA (2006) ABT-089, a neuronal nicotinic receptor partial agonist, for the treatment of attention-deficit/hyperactivity disorder in adults: results of a pilot study. Biol Psychiatry 59:1065–1070 |
| Wilens 2008a | Wilens TE, Adler LA, Weiss MD, Michelson D, Ramsey JL, Moore RJ, Renard D, Brady KT, Trzepacz PT, Schuh LM, Ahrbecker LM, Levine LR (2008a) Atomoxetine treatment of adults with ADHD and comorbid alcohol use disorders. Drug Alcohol Depend 96:145–154 |
| Wilens 2008b | Wilens TE, Klint T, Adler L, West S, Wesnes K, Graff O, Mikkelsen B (2008b) A randomized controlled trial of a novel mixed monoamine reuptake inhibitor in adults with ADHD. Behav Brain Funct 4:24 |
| Wilens 2010 | Wilens TE, Hammerness P, Martelon M, Brodziak K, Utzinger L, Wong P (2010) A controlled trial of the methylphenidate transdermal system on before-school functioning in children with attention-deficit/hyperactivity disorder. J Clin Psychiatry 71:548–556 |
| Wilens 2011a and 2011b | Wilens TE, Gault LM, Childress A, Kratochvil CJ, Bensman L, Hall CM, Olson E, Robieson WZ, Garimella TS, Abi-Saab WM, Apostol G, Saltarelli MD (2011) Safety and efficacy of ABT-089 in pediatric attention-deficit/hyperactivity disorder: results from two randomized placebo-controlled clinical trials. J Am Acad Child Adolesc Psychiatry 50:73-84.e1 |
| Wilens 2015 | Wilens TE, Robertson B, Sikirica V, Harper L, Young JL, Bloomfield R, Lyne A, Rynkowski G, Cutler AJ (2015) A Randomized, Placebo-Controlled Trial of Guanfacine Extended Release in Adolescents With Attention-Deficit/Hyperactivity Disorder. J Am Acad Child Adolesc Psychiatry 54:916-25.e2 |
| Young 2011 | Young JL, Sarkis E, Qiao M, Wietecha L (2011) Once-daily treatment with atomoxetine in adults with attention-deficit/hyperactivity disorder: a 24-week, randomized, double-blind, placebo-controlled trial. Clin Neuropharmacol 34:51–60 |

# **Supplementary Figure S3: Density plots**

Density of the distribution of the variable with missing data (black line) and of the variable with imputed missing data (blue line): SD of placebo effect (top, left), number of centers (top right), sex (middle, left), race (middle, right), ADHD baseline severity (bottom left), and dosification (bottom, right).

| **** |  | **** |
| --- | --- | --- |
| **** |  | **** |
| **** |  | **** |
|  |  |  |

# **Supplementary Figure S4: Funnel plot**

# **Supplementary Table S5: Comparison of the distribution of the study outcome and covariates between the train and the test dataset**

NA = Not Applicable because this characteristic is a binary one (Yes/No)

|  | Train dataset | | | | Test dataset | | | |
| --- | --- | --- | --- | --- | --- | --- | --- | --- |
|  | Mean / % studies | | Min | Max | Mean / % studies | | Min | Max |
| **Study outcome** |  |  | |  |  |  | |  |
| Placebo response | -9,0 | -21,2 | | 2,0 | -8,6 | -15,0 | | 1,3 |
| **Study design-related covariates** |  |  | |  |  |  | |  |
| Design (parallel) | 93,8 | NA | | NA | 89,7 | NA | | NA |
| Naive as inclusion criterion | 6,2 | NA | | NA | 13,8 | NA | | NA |
| Comorbidity as an inclusion criterion | 15,4 | NA | | NA | 13,8 | NA | | NA |
| Number of centres | 23,6 | 1,0 | | 71,0 | 23,0 | 1,0 | | 70,0 |
| Probability of receiving placebo | 38,8 | 10,8 | | 58,6 | 38,7 | 14,8 | | 51,5 |
| ITT analysis | 78,5 | NA | | NA | 86,2 | NA | | NA |
|  |  |  | |  |  |  | |  |
| **Patient-related covariates** |  |  | |  |  |  | |  |
| Age | 23,3 | 8,5 | | 41,4 | 19,2 | 5,1 | | 38,6 |
| Sex (% men) | 64,8 | 27,7 | | 100,0 | 64,2 | 42,9 | | 83,7 |
| Race (% White) | 70,1 | 0,0 | | 98,2 | 74,0 | 0,0 | | 100,0 |
| Baseline ADHD severity | 38,5 | 30,4 | | 46,9 | 38,9 | 34,4 | | 43,5 |
|  |  |  | |  |  |  | |  |
| **Intervention-related covariates** |  |  | |  |  |  | |  |
| Type of Drug (psychostimulant) | 38,5 | NA | | NA | 27,6 | NA | | NA |
| Approval status (Drug approved for treating ADHD) | 80,0 | NA | | NA | 65,5 | NA | | NA |
| Dosification (Fixed) | 41,5 | NA | | NA | 55,2 | NA | | NA |
| Treatment length (weeks) | 8,0 | 2,0 | | 26,0 | 7,7 | 2,0 | | 28,0 |
| Concomitant psychotherapy administered (Yes) | 4,6 | NA | | NA | 0,0 | NA | | NA |
|  |  |  | |  |  |  | |  |
| **Other covariates** |  |  | |  |  |  | |  |
| Publication date | 2011 | 2001 | | 2020 | 2011 | 2001 | | 2019 |
| Study location (USA) | 86,2 | NA | | NA | 86,2 | NA | | NA |
| Sponsor (pharmaceutical industry) | 92,3 | NA | | NA | 93,1 | NA | | NA |
| Risk of bias (High) | 29,2 | NA | | NA | 13,8 | NA | | NA |
|  |  |  | |  |  |  | |  |

# **Supplementary Table S6: Univariate meta-regression results using the training dataset**

|  | **Effect (SE)** | **p-value** |
| --- | --- | --- |
| **Study design-related covariates** |  |  |
| Design (parallel) | -4.14 (2.14) | 0.0525 |
| Naive as inclusion criterion | 1.93 (2.01) | 0.3374 |
| Comorbidity as an inclusion criterion | -0.37 (1.41) | 0.7939 |
| Number of centres | -0.06 (0.02) | 0.0262 |
| Probability of receiving placebo | 0.01 (0.04) | 0.7202 |
| ITT analysis | 1.55 (1.19) | 0.1946 |
|  |  |  |
| **Patient-related covariates** |  |  |
| Age | 0.02 (0.04) | 0.6922 |
| Sex (% men) | -0.02 (0.04) | 0.6310 |
| Race (% White) | 0.02 (0.02) | 0.1949 |
| Baseline ADHD severity | -0.06 (0.14) | 0.6898 |
|  |  |  |
| **Intervention-related covariates** |  |  |
| Type of Drug (psychostimulant) | -0.76 (1.01) | 0.4561 |
| Approval status (Drug approved for treating ADHD) | -0.99 (1.27) | 0.4384 |
| Dosification (Fixed) | -0.28 (1.01) | 0.7795 |
| Treatment length (weeks) | -0.15 (0.11) | 0.1770 |
| Concomitant psychotherapy administered (Yes) | -5.77 (2.36) | 0.0145 |
|  |  |  |
| **Other covariates** |  |  |
| Publication date (years) | -0.33 (0.07) | <.0001 |
| Study location (USA) | -1.06 (1.44) | 0.4642 |
| Sponsor (pharmaceutical industry) | 5.32 (1.88) | 0.0045 |
| Risk of bias (High) | 1.38 (1.09) | 0.2053 |
|  |  |  |

# **Supplementary Table S7: Multivariate meta-regression results using the training dataset**

|  | **Effect (SE)** | **p-value** |
| --- | --- | --- |
| Intercept | 19.12 (9.46) | 0.0432 |
| Design (parallel) | -2.88 (1.88) | 0.1237 |
| Number of centres | -0.03 (0.03) | 0.1625 |
| Concomitant psychotherapy administered (Yes) | -1.31 (2.44) | 0.5915 |
| Publication date (years) | -0.29 (0.07) | 0.0016 |
| Sponsor (pharmaceutical industry) | 7.15 (1.99) | 0.0003 |
|  |  |  |
| tau = 2.6613; I^2^ = 80.84%; R^2^ = 47.29% |  |  |

# **Supplementary Table S8: MetaForest analysis results using the training dataset**

|  | **Importance** | **Direction** | **p-value** |
| --- | --- | --- | --- |
| **Study design-related covariates** |  |  |  |
| Design (parallel) | 0.53 | Negative monotonous | 0.0297 |
| Naive as inclusion criterion |  |  |  |
| Comorbidity as an inclusion criterion |  |  |  |
| Number of centres | 0.32 | Mostly negative | 0.4158 |
| Probability of receiving placebo | -0.09 | Other | 0.7129 |
| ITT analysis |  |  |  |
| **Patient-related covariates** |  |  |  |
| Age | 0.88 | Other | 0.2079 |
| Sex (% men) | 0.96 | Other | 0.1881 |
| Race (% White) | 3.16 | Mostly positive | 0.0099 |
| Baseline ADHD severity | -0.05 | Other | 0.5842 |
| **Intervention-related covariates** |  |  |  |
| Type of Drug (psychostimulant) |  |  |  |
| Approval status (Drug approved for treating ADHD) |  |  |  |
| Dosificaction (% Fixed dosification) |  |  |  |
| Treatment length (weeks) | 0.03 | Mostly negative | 0.4752 |
| Concomitant psychotherapy administered (% with psychotherapy) |  |  |  |
| **Other covariates** |  |  |  |
| Publication date | 3.45 | Mostly negative | 0.0099 |
| Study location (USA) | 0.58 | Negative monotonous | 0.0297 |
| Sponsor (pharmaceutical industry) |  |  |  |
| Risk of bias (High) |  |  |  |

# **Supplementary Table S9: Summary of the results of the MetaForest analysis using the training dataset**

| Publication date *  Race (% White) *  Sex (% men)  Age  Study location (USA) *  Design (parallel) *  Number of centres  Treatment length (weeks)  Baseline ADHD severity  Probability of receiving placebo  *: p-value < 0.05 | 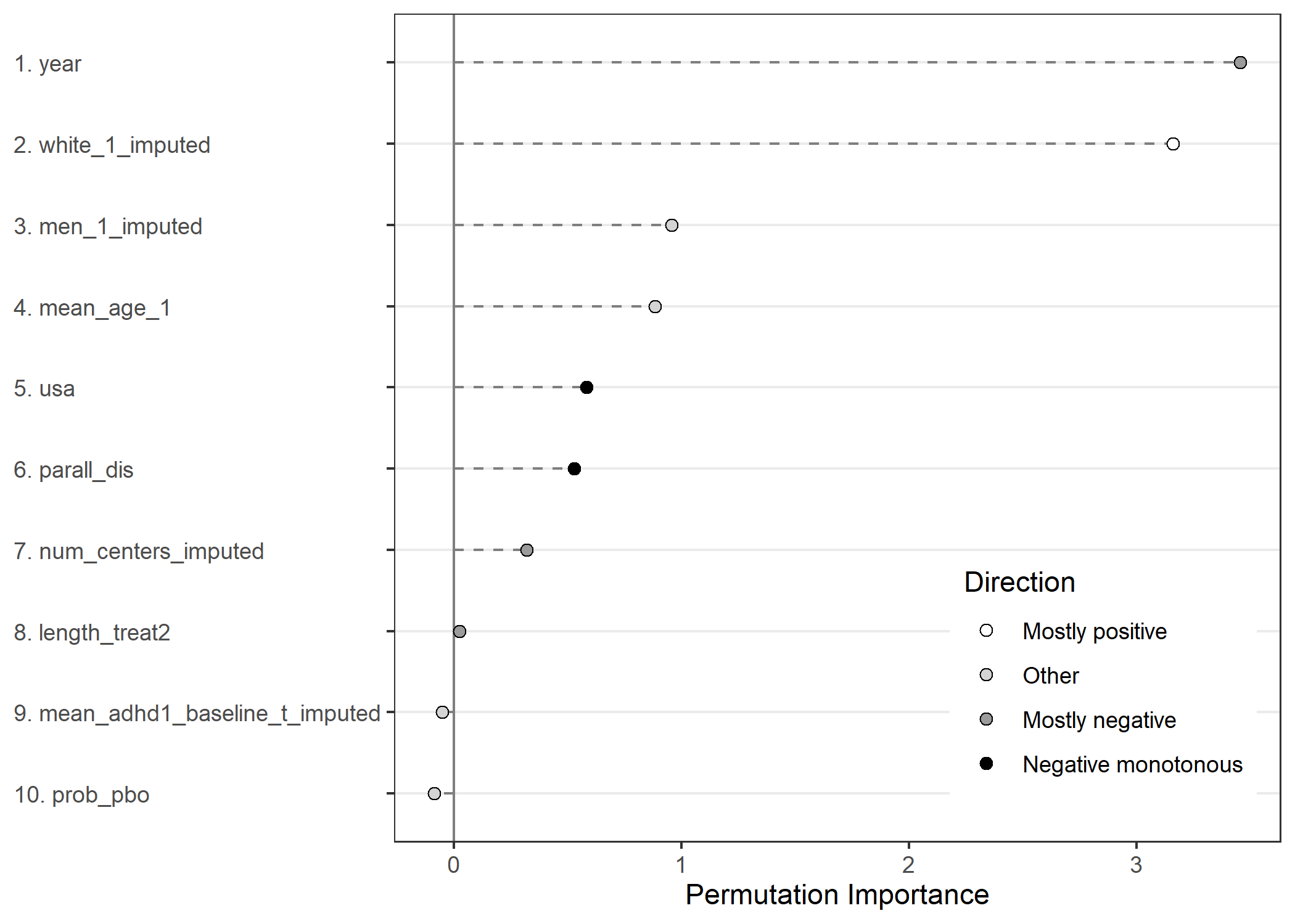 |
| --- | --- |

# **Supplementary Table S10: Univariate meta-regression using a random effects model using the whole dataset**

|  | **Effect (SE)** | **p-value** |
| --- | --- | --- |
| **Study design-related covariates** |  |  |
| Design (parallel) | -3.66 (1.53) | 0.0168 |
| Naive as inclusion criterion | 0.93 (1.37) | 0.4962 |
| Comorbidity as an inclusion criterion | 0.70 (1.12) | 0.5354 |
| Number of centres | -0.07 (0.02) | 0.0006 |
| Probability of receiving placebo | 0.04 (0.03) | 0.2228 |
| ITT analysis | 0.91 (1.00) | 0.3660 |
|  |  |  |
| **Patient-related covariates** |  |  |
| Age | 0.00 (0.03) | 0.9116 |
| Sex (% men) | -0.01 (0.03) | 0.7561 |
| Race (% White) | 0.02 (0.01) | 0.2941 |
| Baseline ADHD severity | -0.10 (0.12) | 0.4187 |
|  |  |  |
| **Intervention-related covariates** |  |  |
| Type of Drug (psychostimulant) | -0.60 (0.81) | 0.4587 |
| Approval status (Drug approved for treating ADHD) | -0.38 (0.92) | 0.6777 |
| Dosificaction (Fixed) | -0.10 (0.78) | 0.8987 |
| Treatment length (weeks) | -0.01 (0.09) | 0.9510 |
| Concomitant psychotherapy administered (Yes) | -5.87 (2.24) | 0.0090 |
|  |  |  |
| **Other covariates** |  |  |
| Publication date (years) | -0.29 (0.07) | <0.0001 |
| Study location (USA) | -2.12 (1.11) | 0.0565 |
| Sponsor (pharmaceutical industry) | 3.40 (1.52) | 0.0255 |
| Risk of bias (High) | 0.97 (0.91) | 0.2880 |
|  |  |  |

# **Supplementary Table S11: Summary of the results of the MetaForest analysis using the whole dataset**

|  | **Importance** | **Direction** | **p-value** |
| --- | --- | --- | --- |
| **Patient-related covariates** |  |  |  |
| Age | 1.02 | Other | 0.0099 |
| Sex (% men) | 1.34 | Mostly negative | 0.1881 |
| Race (% White) | 1.82 | Mostly positive | 0.0990 |
| Baseline ADHD severity | -0.23 | Other | 0.7326 |
| **Intervention-related covariates** |  |  |  |
| Type of Drug (psychostimulant) |  |  |  |
| Concomitant psychotherapy administered (Yes) |  |  |  |
| Approval status (Drug approved for treating ADHD) |  |  |  |
| **Study design-related covariates** |  |  |  |
| Design (parallel) | 0.11 | Negative monotonous | 0.1485 |
| Naive as inclusion criterion |  |  |  |
| Number of centres | 0.67 | Mostly negative | 0.1584 |
| Dosificaction (Fixed) |  |  |  |
| Treatment length (weeks) | -0.31 | Mostly negative | 0.8514 |
| Probability of receiving placebo | -0.20 | Other | 0.8118 |
| Comorbidity as an inclusion criterion |  |  |  |
| ITT analysis |  |  |  |
| **Other covariates** |  |  |  |
| Publication date | 2.89 | Mostly negative | 0.0099 |
| Study location (USA) | 0.53 | Negative monotonous | 0.0198 |
| Sponsor (pharmaceutical industry) |  |  |  |
| Risk of bias (High) |  |  |  |
